# Quickly identifying identical and closely related subjects in large databases using genotype data

**DOI:** 10.1371/journal.pone.0179106

**Published:** 2017-06-13

**Authors:** Yumi Jin, Alejandro A. Schäffer, Stephen T. Sherry, Michael Feolo

**Affiliations:** National Center for Biotechnology Information, National Library of Medicine, National Institutes of Health, Bethesda, Maryland, United States of America; Universitat Pompeu Fabra, SPAIN

## Abstract

Genome-wide association studies (GWAS) usually rely on the assumption that different samples are not from closely related individuals. Detection of duplicates and close relatives becomes more difficult both statistically and computationally when one wants to combine datasets that may have been genotyped on different platforms. The dbGaP repository at the National Center of Biotechnology Information (NCBI) contains datasets from hundreds of studies with over one million samples. There are many duplicates and closely related individuals both within and across studies from different submitters. Relationships between studies cannot always be identified by the submitters of individual datasets. To aid in curation of dbGaP, we developed a rapid statistical method called Genetic Relationship and Fingerprinting (GRAF) to detect duplicates and closely related samples, even when the sets of genotyped markers differ and the DNA strand orientations are unknown. GRAF extracts genotypes of 10,000 informative and independent SNPs from genotype datasets obtained using different methods, and implements quick algorithms that enable it to find all of the duplicate pairs from more than 880,000 samples within and across dbGaP studies in less than two hours. In addition, GRAF uses two statistical metrics called All Genotype Mismatch Rate (AGMR) and Homozygous Genotype Mismatch Rate (HGMR) to determine subject relationships directly from the observed genotypes, without estimating probabilities of identity by descent (IBD), or kinship coefficients, and compares the predicted relationships with those reported in the pedigree files. We implemented GRAF in a freely available C++ program of the same name. In this paper, we describe the methods in GRAF and validate the usage of GRAF on samples from the dbGaP repository. Other scientists can use GRAF on their own samples and in combination with samples downloaded from dbGaP.

## Introduction

Genome-wide association studies (GWAS) have identified numerous associations between genotypes and complex clinical conditions and phenotypic traits. Thousands of research articles have been published; more than 20,000 single nucleotide polymorphisms (SNPs) have been identified to be associated with various human phenotypes and diseases [1]. Knowledge of any close relationships between individuals is crucial to the validity of the analysis results. Closely related individuals should not be used together in case-control and population based association studies [2–5]. Cryptic relatedness can increase both false positive and false negative rates [4,6]. Although genetic relatedness can be calculated from reported pedigree structures, these structures are often incomplete and incorrect due to unreported relationships, non-paternity, adoption or sample labeling errors [7,8]. Fortunately, pedigree errors and cryptic relatedness can be detected using genotype data obtained in GWAS studies.

Algorithms and software packages have been developed to determine the subject relationships using genotype data [2,9–17]. Most of the existing methods determine genetic relationships by estimating the genome-wide averages of the number of alleles shared identical by descent (IBD) and/or kinship coefficient between each pair of individuals, with computation complexities at least *O(n*^*2*^*S)*, where *n* is the number of samples, and *S* is the number of SNPs. These algorithms were not optimized to determine subject relationships in datasets with more than a few thousand samples. However, the establishment of centralized databases such as the database of Genotypes and Phenotypes (dbGaP) at the National Center for Biotechnology Information (NCBI) and the European Genome-phenome Archive (EGA) at European Bioinformatics Institute has made it possible for researchers to access genotypic data from millions of individuals.

Two novel aspects arise with large controlled access databases. First, users will obtain permission to access only subsets of the data. Second, different users will have access to different subsets. Therefore, it is desirable for the database owners to *precompute* all possible relationships among all datasets. Then, each user can be provided with the precomputed relationships among only those datasets retrieved. The duplicates and close relationships should be determined even when the individual datasets have genotypes obtained from different genotyping platforms.

Two types of methods are commonly used to determine genetic relationships between individuals based on molecular markers: maximum-likelihood estimation (MLE) [18–20] and method-of-moment estimation (MM) [8,13,21,22]. Both MLE and MM methods use the observed numbers of alleles shared identity by state (IBS) to estimate the IBD sharing probabilities or kinship coefficients. Milligan [23] compared the statistical performance of these two types of methods. The maximum-likelihood estimators always generate biologically meaningful probabilities and are usually more accurate than the method-of-moment estimators. However, maximum-likelihood approaches are usually slower and sometimes more biased than the method-of-moment ones. Method-of-moment methods usually use the observed numbers of IBS sharing loci instead of the predicted numbers when calculating IBD sharing probabilities, and hence may yield estimates that cannot be interpreted as probabilities, in which case, researchers truncate the estimates into the meaningful range [0, 1]. The truncation of the results introduces artificial effects and biases.

Besides the computational complexity, another limitation of the existing algorithms is that they can be applied only to genotype datasets obtained using the same genotyping method (i.e. various SNP arrays or sequencing assays). For a genotype repository, such as dbGaP, the submitted genotype datasets have been obtained using many different genotyping methods.

We have developed algorithms to extract genotypes of common SNPs from genotype datasets obtained using different methods, and developed these algorithms into a software package called GRAF (Genetic Relationship and Fingerprinting) to determine quickly the relationships between subjects originally genotyped in disparate studies. The algorithms and software described in this article were developed as quality control tools for dbGaP curators to check all studies prior to release, and find subject overlaps across dbGaP studies. GRAF can also be used by dbGaP submitters and users to find errors in the genotype datasets and pedigree files.

The GRAF algorithms and software were briefly presented at the 22nd Annual International Conference on Intelligent Systems for Molecular Biology [24] and the 64th Annual Meeting of the American Society of Human Genetics [25] in 2014. Later, the KING software [11] was upgraded to version 2.0 to handle more than a million samples [26]. Since KING is most similar to our methods and can handle large data sets, we focus our comparisons on KING 2.0, using real genotype data with very large sample sizes.

## Results

### dbGaP and genotype fingerprinting

NCBI’s dbGaP database is a repository charged to archive, curate, and distribute information produced by genome scale studies investigating the interaction of genotypes and phenotypes [27]. As of August 15, 2016, the genotype and/or phenotype data of 1,430,765 samples collected from 1,091,830 research participants (referred to as subjects in this article) across 671 studies have been released to researchers by dbGaP.

Samples from a single subject or from closely related subjects are often submitted to different studies without being reported by dbGaP submitters, who may not know if the subjects participated in previously submitted studies. The dbGaP processing pipeline identifies samples that originate from the same subject and assigns a unique resource-wide subject ID (a.k.a., dbGaP Subject ID) for any genotyped subject. Theoretically, a few dozen genotypes from informative and independent SNPs can be used as “fingerprints” to distinguish subjects from each other in a database with millions of samples [28,29]. However, the genotypes submitted to dbGaP are obtained with different methods (i.e., various arrays or sequencing) and there are no SNPs that are covered by all the genotyping methods.

To support the dbGaP processing pipeline, we curated a large collection of markers that could be used for identity and relationship testing, which we refer to as the *dbGaP Fingerprinting Collection*. This collection was chosen to increase the chance that each sample has enough genotype information to identify it, while keeping the number of markers bounded. We selected 10,000 autosomal SNPs, where each had the following properties: (1) appears on at least 80% of the genotyping platforms thus far submitted to dbGaP; (2) is bi-allelic; (3) has minor allele frequency (MAF) > 0.17 as reported by the 1000 Genomes Project [30]; (4) is separated from other selected SNPs by at least 50,000 bps; and (5) contains no complementary alleles, i.e., no A/T or G/C alleles. The non-complementary rule was imposed to avoid the DNA strand orientation problem. For example, if the two alleles of a fingerprinting SNP are A and G, then we know that the genotype AA in one dataset is the same as genotype TT in another dataset obtained with different genotyping methods, eliminating the need to know the DNA strand orientation used. If instead the alleles were A and T, then the meaning of genotypes AA and TT could not be disambiguated without knowing the DNA strand orientations.

We extracted the genotypes of 10,000 fingerprinting SNPs from binary PLINK.bed files [31] for 860,211 samples from 286 dbGaP studies and built the dbGaP Fingerprinting Collection. Each sample had at least 1,000 fingerprinting SNPs genotyped (Supporting Information, S1 Table). The average genotype missing rate resulting from SNPs not being on the chips or platforms is 9.1%, and the average rate of missing genotypes not called by genotyping methods is 2.9%.

### Finding identical samples: IBS, IBD and subject relationships

The genetic relatedness between two subjects can be estimated from SNP genotypes as a kinship coefficient—the probability that two alleles at the same locus are identical by descent (IBD). Some high values of the kinship coefficient are associated with specific relationships (e.g., second degree relatives). An important exception is that parent-offspring and full siblings have the same kinship coefficient of 0.25. The probability distributions of IBD cardinality *P(Z = 0*,*1*,*2)*, where *Z* is the number of alleles IBD, are more informative than kinship coefficients. Here, we restrict our analysis to relationships no more distant than third degree relatives.

**Fig 1:**
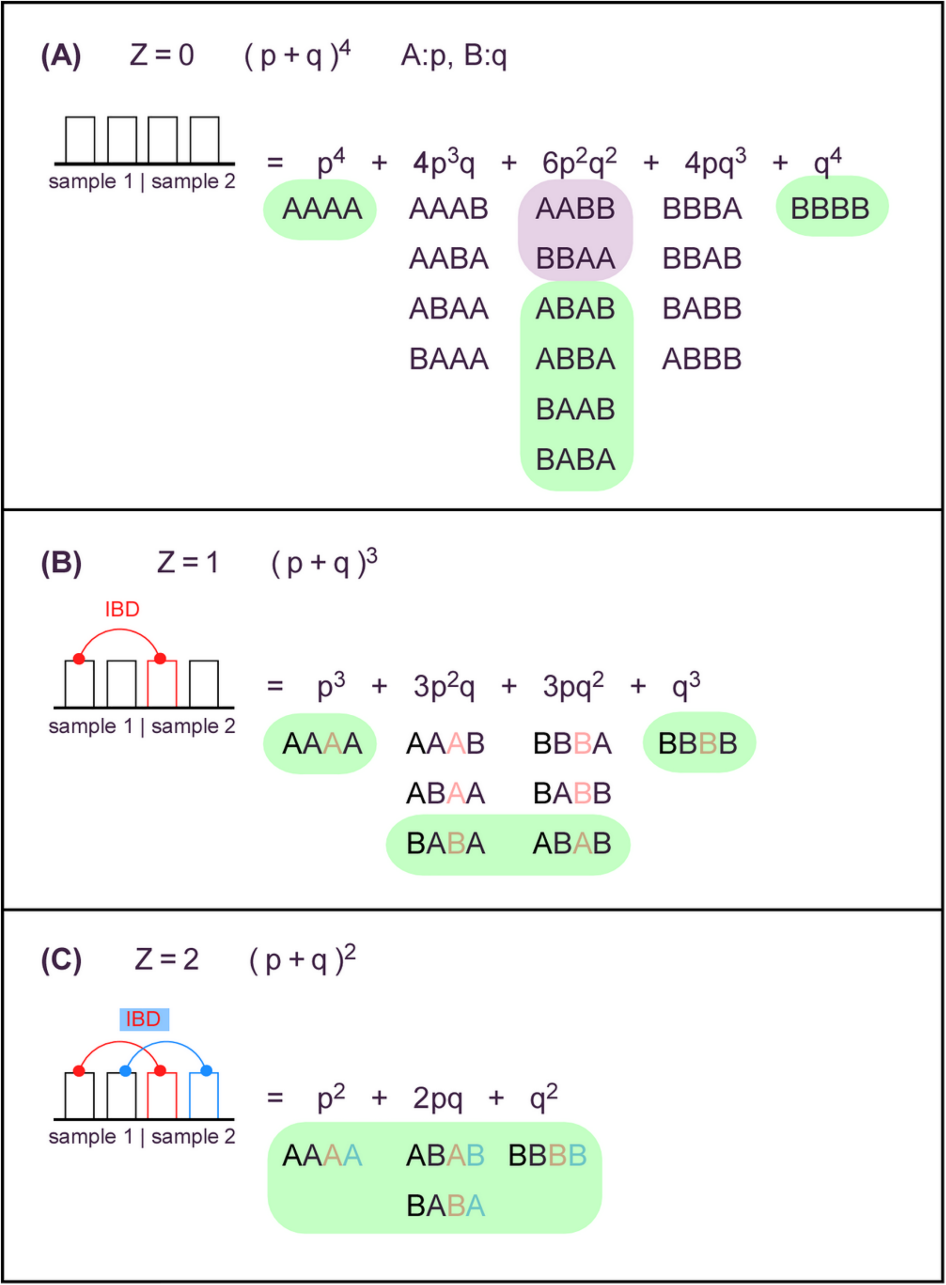
Probabilities of shared alleles in pairwise sample comparisons for autosomal bi-allelic markers are derived from the list of genotype outcomes. When no alleles are shared by descent (*Z*) (panel A, *Z* = 0), then the chance of seeing any specific combination of alleles is the product of the respective allele frequencies. When one (panel B, *Z* = 1) or both alleles (panel C, *Z* = 2) are shared by descent, then the possible number of genotype outcomes are reduced. The number of alleles identical by state (*I*) can be zero (panel A, lavender), one (all panels, no highlight), or two (all panels, green).

Suppose a homogeneous, random mating population in Hardy-Weinberg equilibrium, having a bi-allelic SNP at location *i* with allele frequencies *p*_*i*_ and *q*_*i*_. Any two subjects in the population can be observed to share 0, 1, or 2 alleles identical by state (IBS). Some of the IBS alleles shared by two subjects might be inherited from the same recent ancestor, or identical by decent (IBD). If we denote the number of IBD alleles as *Z* and the number of IBS alleles as *I*, as used by Purcell et al[13], the probability of each IBS state given an IBD state, *P(I|Z)* can be calculated by using the formulas in Table 1. Fig 1 shows how the equations in Table 1 are derived. For a pair of subjects, the kinship coefficient *ϕ* can be calculated using the *P(Z)* values: *ϕ = P(Z = 2)/2 + P(Z = 1)/4*.

**Table 1:** Calculation of *P(I|Z)* value for each marker.

	IBD state		
IBS state	*Z = 0*	*Z = 1*	*Z = 2*
*I = 0*	*$2p_{i}^{2}q_{i}^{2}$*	0	0
*I = 1*	*$4p_{i}^{3}q_{i}+4p_{i}q_{i}^{3}$*	*$2p_{i}^{2}q_{i}+2p_{i}q_{i}^{2}$*	0
*I = 2*	*$p_{i}^{4}+q_{i}^{4}+4p_{i}^{2}q_{i}^{2}$*	*$p_{i}^{3}+q_{i}^{3}+p_{i}^{2}q_{i}+p_{i}q_{i}^{2}$*	1

### Statistical metrics to determine subject relationships

Assuming there is no inbreeding, the probabilities of different IBD states for common types of relationships are shown in the left columns in Table 2. The results in Table 2 indicate that we can use *P(Z = 2)* to distinguish identical pairs (including samples from the same subject and those from monozygotic twins) from other types of relationships. We can also use *P(Z = 0)* to distinguish other types of close relationships.

**Table 2:** Probabilities of different IBD and IBS states for different relationships.

Relationship	Abbrev.	*P(Z = 0)*	*P(Z = 1)*	*P(Z = 2)*	*P(I<2)*
Identical pair	ID	0	0	1	0.00
Parent-offspring	PO	0	1	0	0.39
Full sibling	FS	¼	½	¼	0.33
Second degree	D2	½	½	0	0.47
Third degree	D3	¾	¼	0	0.50
Unrelated	UN	1	0	0	0.54

We compare the SNP genotypes of every two samples and use two statistical metrics to find their relationships. The first one is the genotype discordance rate when all SNPs with genotypes are counted, which we call All Genotype Mismatch Rate (AGMR), equivalent to the percentage of SNPs with IBS state *I < 2*. The second one is the genotype mismatch rate when only the SNPs with both samples being homozygous are counted, which we call Homozygous Genotype Mismatch Rate (HGMR). Assuming a homogenous, random mating population, with allele frequencies uniformly distributed in the range between 0.1 and 0.9, the expected AGMR values are the same as the *P(I<2)* values in Table 2, and the expected HGMR values and standard deviations are shown in Table 3.

**Table 3:** Predicted HGMR values and standard deviations for different types of relationships assuming allele frequencies are evenly distributed between 0.1 and 0.9.

Relationship	Expected HGMR	SD
Parent-offspring	0.0000	0.0000
Full sibling	0.0454	0.0031
Second degree	0.1040	0.0050
Third degree	0.1594	0.0062
Unrelated	0.2172	0.0073

### Identical pairs within and between dbGaP studies

To find identical pairs within and across studies, we have developed three different algorithms—the naïve, quadratic and sub-quadratic—with computational complexities *O(n*^*2*^*S)*, *O(n*^*2*^*)*, and *O(n log n)*, respectively (see Methods). The naïve algorithm and the quadratic algorithm tolerate high genotype missing rates in the genotype datasets, but the sub-quadratic algorithm requires low genotype missing rates.

The naïve and the quadratic algorithms were implemented as multithreaded programs. We tested the naïve algorithm, the quadratic algorithm, and the duplicate sample detection function of KING 2.0 using the genotype dataset extract from all dbGaP studies on August 15, 2016, with minimum 1000 fingerprint SNPs having genotypes, on an Intel Xeon machine with 16 2.67 GHz CPUs. This dataset contains 884,794 samples, with average genotype missing rate 11.7%.

It took the naïve algorithm 65 hr 42 min to check all the samples. The running time of quadratic algorithm was 1 hr 34 min. KING 2.0, when being run with option “-duplicate” to only find duplicate samples, took 1 hr 48 min.

Since the native algorithm exhaustively checks all pairs of samples over all SNPs, we can use the identical pairs found by the naïve algorithm to evaluate the accuracies of the other two programs. Also since both the quadratic algorithm and KING 2.0 check genotypes of all SNPs at the last step, the specificities of both algorithms are 100%. Assuming all sample pairs with AGMR less than 10% are real identical pairs, Table 4 compares the sensitivities of these two algorithms for finding identical pairs with different AGMR ranges.

**Table 4:** Comparison of the performances of the GRAF quadratic algorithm and KING 2.0 on finding identical pairs with different AGMR ranges.

		GRAF		KING			
AGMR (%)	#ID pairs	#Missed	FN (%)	#Missed	FN (%)	Mean AGMR (%)	Exp. FN (%)
[0, 1)	87500	0	0.00	1102	1.26	0.08	4.1E-13
[1, 2)	1446	0	0.00	237	16.39	1.37	2.7E-04
[2, 3)	276	0	0.00	29	10.51	2.38	0.014
[3, 4)	72	1	1.39	10	13.89	3.45	0.159
[4, 5)	38	0	0.00	9	23.68	4.36	0.656
[5, 6)	15	0	0.00	3	20.00	5.48	2.337
[6, 7)	20	1	5.00	1	5.00	6.44	5.263
[7, 8)	11	1	9.09	2	18.18	7.49	10.450
[8, 9)	8	1	12.50	6	75.00	8.64	18.563
[9, 10)	6	4	66.67	6	100.00	9.40	25.121
[0, 10)	89392	8	0.01	1405	1.57	0.12	1.8E-12

All of the identical pairs found by the naïve algorithm are grouped by AGMR values. #Missed: number of identical pairs missed by the algorithm; FN: false negative rate; Mean AGMR: average AGMR of the identical pairs in this group; Exp. FN: expected false negative rate of the quadratic algorithm calculated using Eq 5 (see Methods) supposing all sample pairs have the mean AGMR within the interval on that row of the table.

Table 4 shows that GRAF quadratic algorithm has a much lower false negative rate than KING 2.0, especially when the genotype discordance rates are high. We also grouped the identical pairs by number of SNPs with genotypes and compared the performances of the two algorithms. Table 5 shows the results. All identical pairs had at least 287 SNPs with genotypes for each samples. GRAF and KING both worked well when more than 4000 SNPs were genotyped for both samples in each pair. When fewer SNPs had genotypes, GRAF could still find almost all of the identical pairs, but KING failed to detect many identical pairs.

**Table 5:** Comparison of the performances of the GRAF quadratic algorithm and KING 2.0 on finding identical pairs with different numbers of SNPs with genotypes.

		GRAF		KING 2.0	
#SNPs with genotypes	Total pairs	#Missed	FN (%)	#Missed	FN (%)
287–1000	282	0	0.00	282	100.00
1001–2000	975	1	0.10	953	97.74
2001–3000	158	0	0.00	42	26.58
3001–4000	929	2	0.22	111	11.95
4001–5000	3743	0	0.00	0	0.00
5001–6000	39	0	0.00	0	0.00
6001–7000	4604	1	0.02	2	0.04
7001–8000	12030	2	0.02	1	0.01
8001–9000	3984	0	0.00	0	0.00
9001–10000	62648	2	0.00	14	0.02
287–10000	89392	8	0.01	1405	1.57

#SNPs with genotypes: Ranges of numbers of SNPs with genotypes for both of the samples in each identical pair

#Non-null SNPs: For each identical pair, non-null SNPs are the SNPs at which both samples have genotypes. This column shows the ranges of counts of non-null SNPs.

We also compared the sub-quadratic algorithm and the quadratic algorithm using all the samples in dbGaP. As mentioned above, comparing to the other two algorithms, the sub-quadratic algorithm is more sensitive to the missing genotype rate than the quadratic algorithm. To evaluate the performance of the sub-quadratic algorithm, we created several datasets from all of dbGaP studies by extracting genotypes of samples with maximum numbers of missing genotypes below seven thresholds: 10, 100, 1000, 2000, 3000, 5000 and 9000. Table 6 shows the results. The sub-quadratic algorithm was single-threaded. The running time against the dataset with 884,787 samples was 15 min. When the quadratic algorithm was run using one CPU core, it took 15 hr 39 min to check all of sample pairs.

**Table 6:** Running times and prediction accuracies of the sub-quadratic algorithm tested with datasets of different sample sizes and genotype missing rates.

Dataset				Performance of sub-quadratic algorithm			
Max geno miss	Mean geno miss	#Samples	Total ID pairs	#Rounds	Time (min)	#FN	FN (%)
10	5.7	68868	6975	12	0.6	0	0.0
100	32.9	407713	44820	45	3.8	0	0.0
1000	153.7	669927	62931	70	6.7	2	0.0
2000	184.5	688125	66766	88	7.2	44	0.1
3000	415.4	755156	81380	112	9.0	9127	11.2
5000	500.8	774255	83379	113	9.4	11240	13.5
9000	1166.5	884794	89392	135	15.3	13432	15.0

The sub-quadratic algorithm was run five times for each input and the results were averaged and shown on this table. Max geno miss: maximum number of SNPs without genotypes for each sample; Mean geno miss: average number of SNPs per sample without genotypes; Total ID pairs: total number of identical pairs in each dataset found by the naïve algorithm; #Rounds: number of rounds ran by the sub-quadratic algorithm before it converged.

Table 6 confirms that the sub-quadratic algorithm performs better when the genotype missing rate is not too high. When the maximum number of missing genotypes per sample is 2000 or fewer (average 185 or fewer missing genotypes), the sub-quadratic algorithm could detect 99.9% or more identical pairs. However, for the dataset with maximum 9000 missing genotypes per sample, it was able to find only 85% of the identical pairs.

The measured performances for the sub-quadratic algorithm were achieved when the two parameters *m* (number of markers checked) and *c* (number of rounds for convergence) were set to 20 and 10, respectively (see Methods for more details on the meanings of *m* and *c*). Increasing *c* can increase the sensitivity, at the cost of longer running time. Another way to increase the sensitivity is to run the sub-quadratic algorithm multiple times and take a union of the identical pairs obtained in different runs.

**Fig 2:**
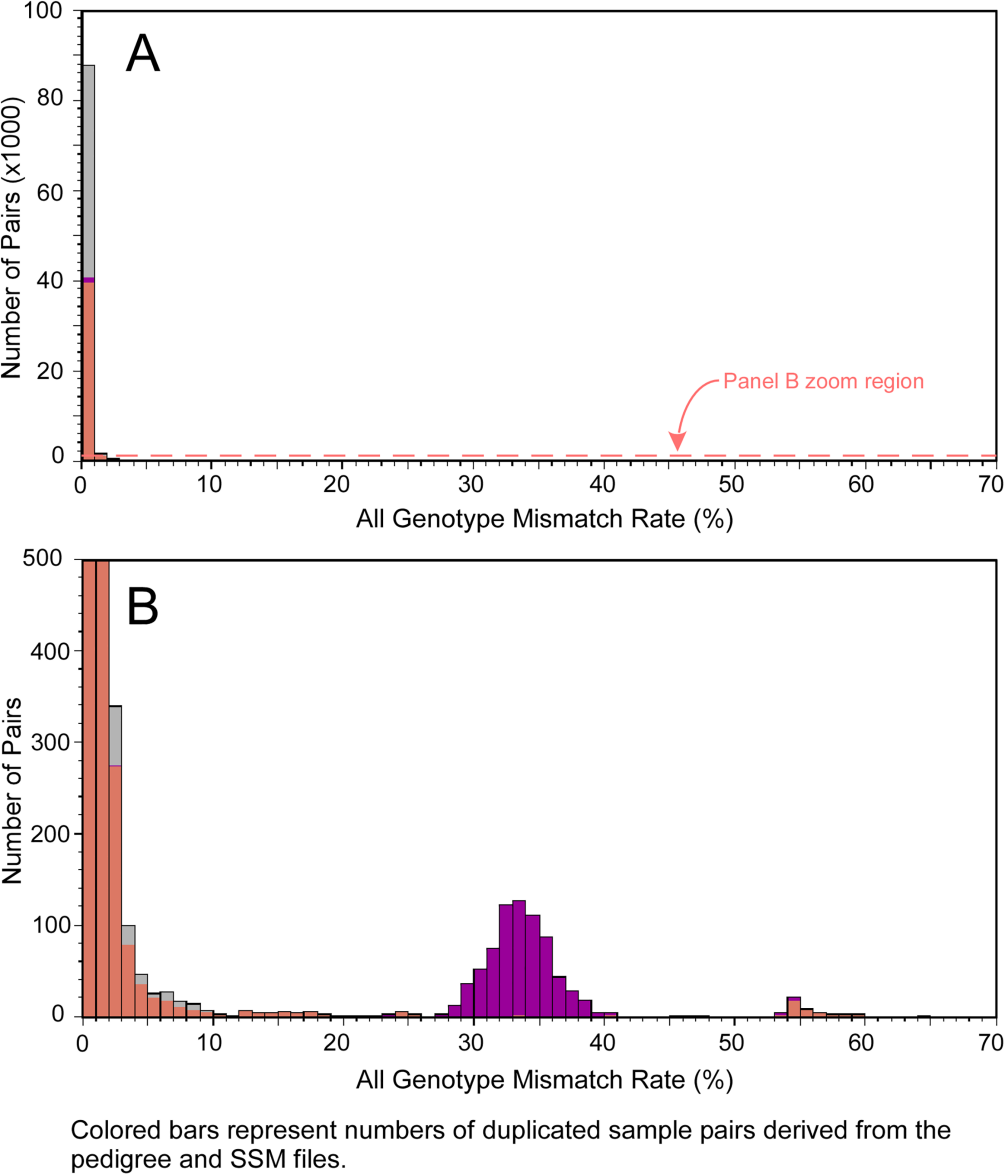
Distribution of all genotype mismatch rates of identical pairs detected by the quadratic algorithm and those reported by submitters. All samples in the dbGaP Fingerprint Collection are compared. Types of submitter-reported relationships are color coded. Coral red: samples are reported to be from the same subjects; Purple: samples are from monozygotic twins; Gray: no relationship reported by submitters. Panel A shows the whole graph. Panel B shows the same graph, stretched on y-axis to show details of the bottom part of the graph.

Since the quadratic algorithm is both fast and robust, tolerating high genotyping errors and systematic genotype gaps resulted from different genotyping methods, it is routinely used by dbGaP curators to find identical pairs within and across studies. We set the AGMR cutoff value to 20% when actually reporting putative identical pairs. For 884,797 samples in the database with maximum 9000 missing genotypes per sample, excluding those sample pairs that are proved to be incorrect by checking the genotypes, there are 42,402 identical sample pairs (24,127 within study and 18,275 across studies) reported by submitters; all 42,402 identical pairs were detected by the quadratic algorithm. In addition, the quadratic algorithm was able to find 47,433 identical pairs not reported by the submitters. Of these, most (46,124) are across studies and hence, would be unrecognizable to the submitters of single studies (Fig 2A).

Fig 2 shows the distribution of the genotype mismatch rates of all the identical pairs detected by the quadratic algorithm, as well as those reported by the submitters. When the same graph is scaled to show the details (Fig 2B), we can see that there is a peak with average AGMR about 33%. Most of the pairs under this peak are reported by submitter as monozygotic twins. However, Table 2 shows that 33% is the expected AGMR value of full sibling pairs. So, it is likely that most of these pairs are non-identical twins reported as identical twins by mistake.

Fig 2A also shows a small peak with average AGMR about 54%, corresponding to the expected AGMR for unrelated pairs, indicating these pairs of samples are actually from unrelated or remotely related subjects, but reported incorrectly by submitters as either from the same subject or identical twins. The sample pairs with low AGMR values, e.g., less than 20%, are most likely collected from same subjects or identical twins. The genotype mismatches are caused by errors in genotyping, data processing, or sample mixups.

### Using HGMR to determine non-identical, closely related pairs of subjects

Table 3 shows that HGMR can be used to distinguish subject pairs with different relationships in a homogeneous, random mating population. Actual human populations are much more complicated than this simple model. We selected some dbGaP studies and tested how HGMR values can be used to determine the subject relationships with real data.

**Fig 3:**
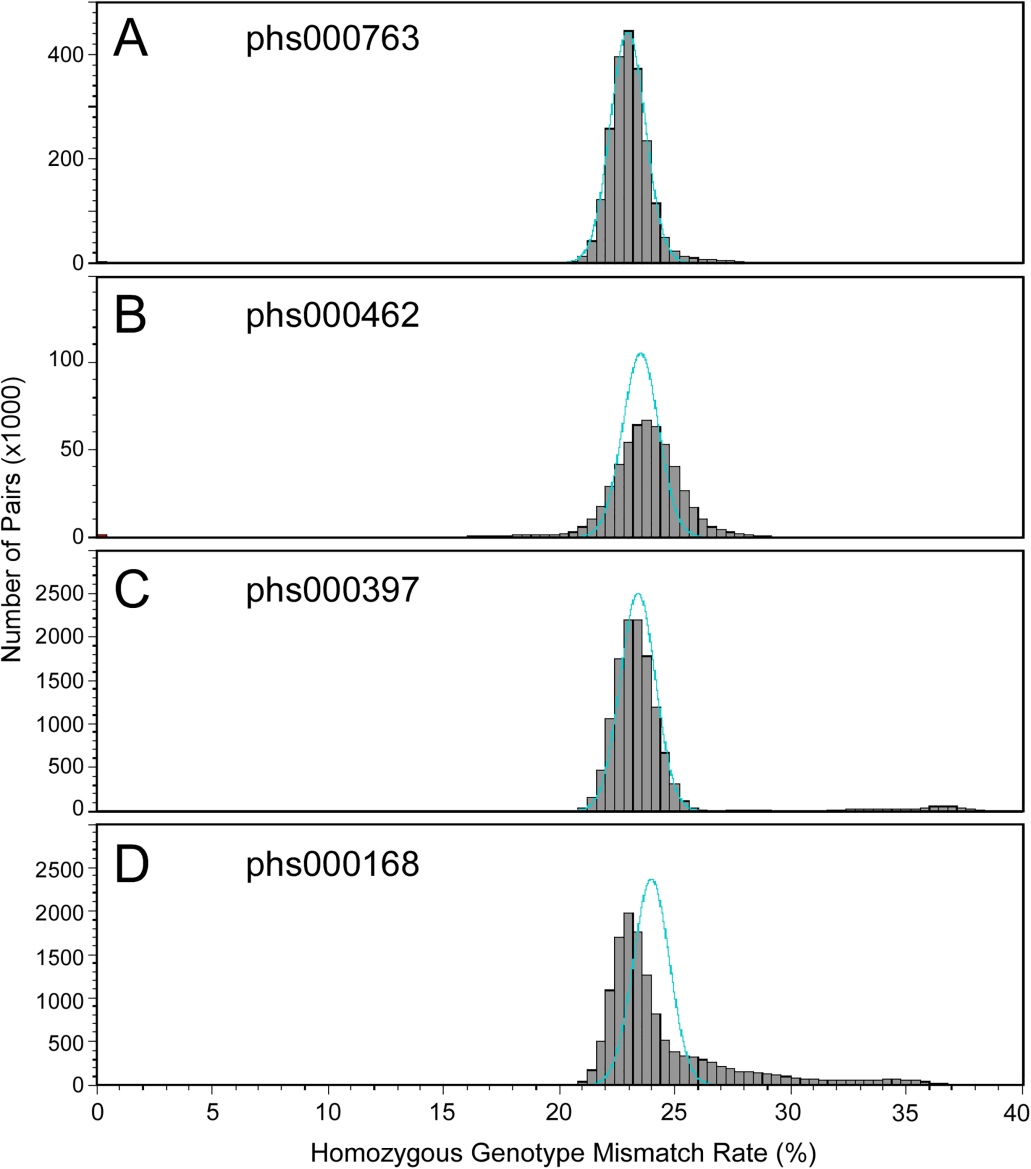
Distribution of homozygous genotype mismatch rates of sample pairs in four dbGaP studies. Cyan curves show the distribution of HGMR values predicted with the assumption that the populations are homogeneous and random mating.

**Fig 4:**
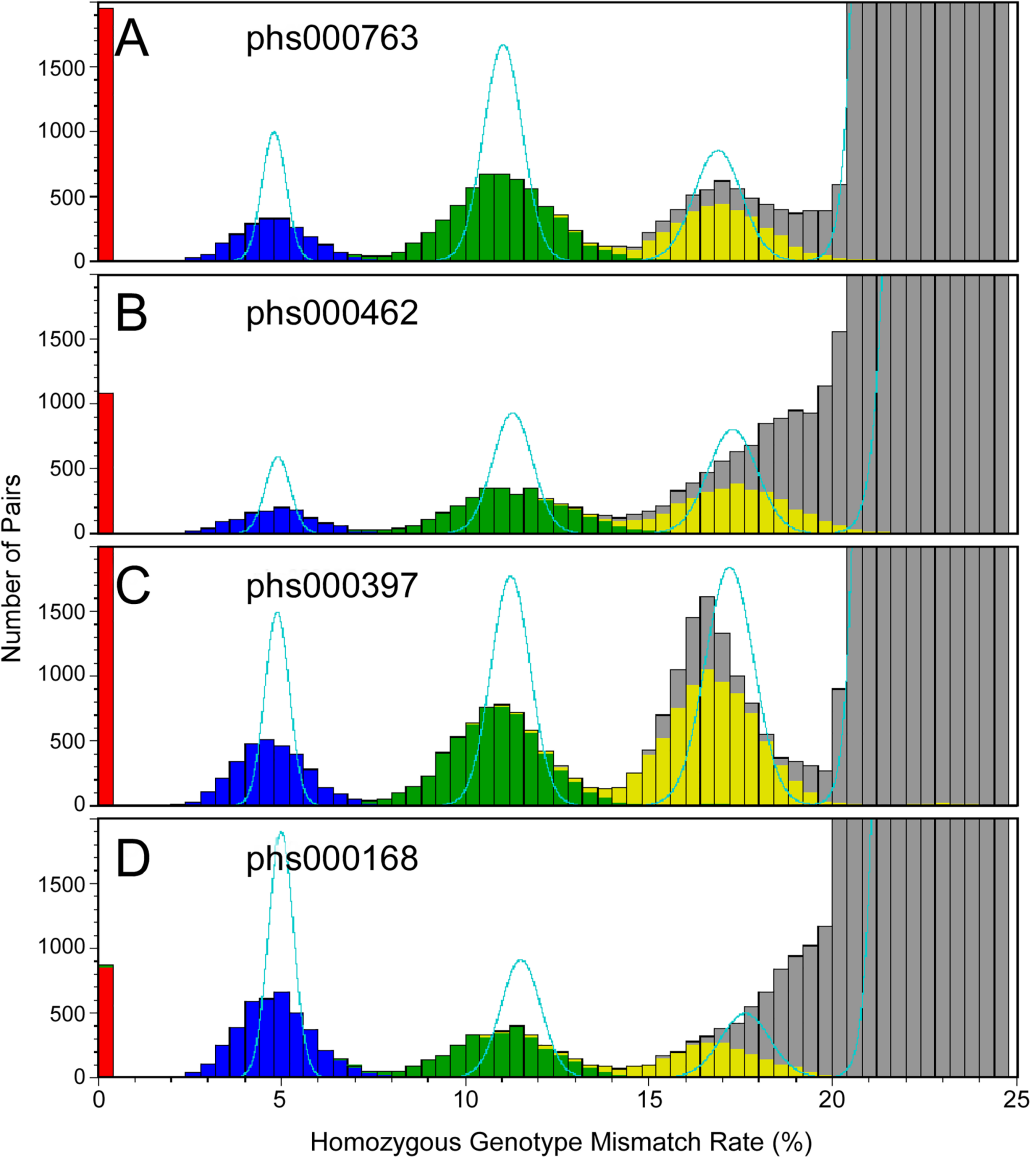
Distribution homozygous genotype mismatch rates of sample pairs in the same four dbGaP studies as in Fig 2. Graph is zoomed in to show the related pairs. Cyan curves show the distribution of HGMR values predicted with the assumption that the populations are homogeneous and random mating. Types of relationships reported by submitters in pedigree files are color coded. Red: parent/offspring; Blue: full sibling; Green: second degree relative; Yellow: third degree relative; Gray: no relationship reported by submitters.

As of August 15, 2016, there were four dbGaP studies with at least 500 pairs of related subjects reported by submitters for each relationship type of full sibling, second degree relatives (including grandparent-grandchild, avuncular, and half sibling pairs) and third-degree relatives (including first cousin and half-avuncular pairs), and with fingerprinting genotype missing rate less than 10% (Table 7). We compared the genotypes of all the fingerprinting SNPs for all the sample pairs within each study using the naïve algorithm (see Methods). For all the studies, the distributions of HGMR values are close to the normal distributions predicted by assuming the populations are homogeneous, random mating (Fig 3). The matches are especially good for studies phs000763 (Fig 3A) and phs000397 (Fig 3C). Fig 4 shows HGMR distributions of the subject pairs of the same studies, scaled and color-coded to display the closely related pairs. The mean HGMR values of full sibling, second degree relative, and third degree relative are close to the predicted values, but the standard deviations are much larger than the values predicted using the simple homogeneous, random mating model. For parent-offspring pairs, the predicted HGMR value is 0, and the actual values are also close to 0.

**Table 7:** Genotype missing rates and numbers of pairs of related samples reported in submitted pedigree files for four dbGaP studies.

			Pairs of related samples reported in pedigree files			
Study	Samples	Geno MR(%)	Parent-offspring	Full sibling	Second degree	Third degree
phs000763	2058	0.59	1947	2028	5607	3535
phs000462	1035	0.81	1079	1228	3211	3500
phs000397	5013	0.17	2332	3119	6027	7878
phs000168	5220	0.12	852	3984	3136	2315

### Using both HGMR and AGMR to determine subject relationships for dbGaP studies

We have shown that AGMR can be used to distinguish identical pairs from other types of relationships, and the latter can be separated from each other by using HGMR. Table 2 also shows that the expected AGMR values are different among different types of relationships, and hence can also be used to separate these relationships, especially to separate full siblings from second degree relatives. To obtain greater discrimination power, we use joint HGMR and AGMR values to determine subject relationships.

If HGMR and AGMR values both follow normal distributions, then the joint values follow a bivariate normal distribution. We estimate some parameters using the genotype data in dbGaP Fingerprinting Collection of subjects with known to be closely related (Table 8), and use the probability density function of the bivariate normal distribution to calculate the prior probabilities to obtain different AGMR and HGMR values for each type of relationship. After excluding the identical pairs and more remotely related subject pairs, we can calculate the posterior probability of any pair of subjects being PO, FS, D2, or D3 given AGMR and HGMR values, using Bayes theorem (see Methods). Using this method, we are able to directly determine subject relationships using SNP genotypes without estimating *P(Z)* values or kinship coefficients.

**Table 8:** Mean HGMR and AGMR values and correlation coefficients between HGMR and AGMR of all related subjects reported in the data files submitted to dbGaP.

		HGMR (%)		AGMR (%)		
Relationship	Number of pairs	Mean	SD	Mean	SD	Correlation coefficient
Identical	21099	0.00	0.00	0.08	0.38	0.317
Parent-offspring	45184	0.04	0.07	39.48	1.47	0.104
Full sibling	28447	4.86	1.02	33.59	2.32	0.784
Second degree	18492	11.16	1.35	47.30	1.10	0.803
Third degree	10841	17.32	1.35	51.07	0.96	0.830

**Fig 5:**
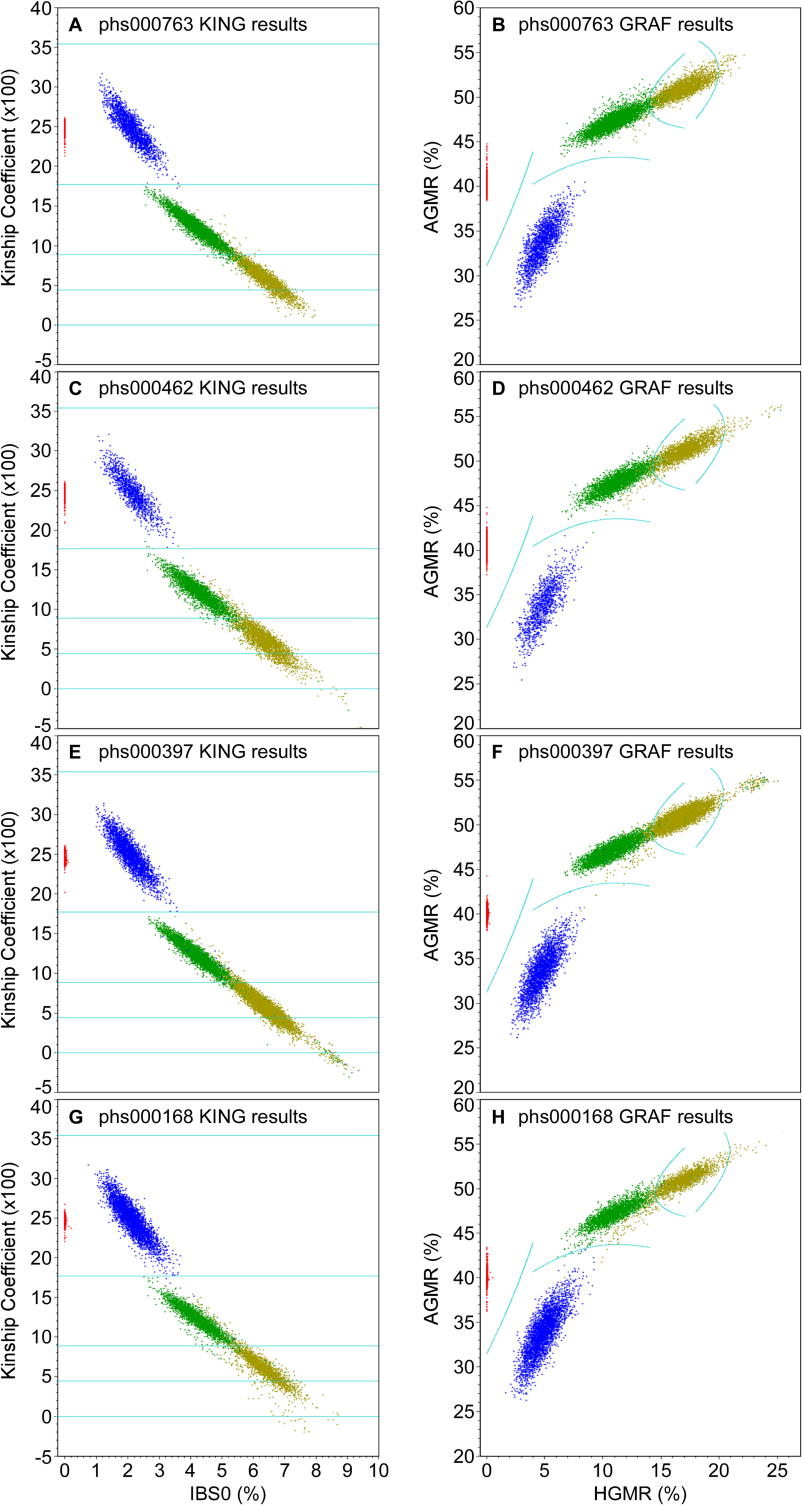
Distribution of both HGMR and AGMR values of all pairs of samples reported by submitters as closely related. Each dot represents one pair of samples. Types of relationships reported by submitters are color coded. Purple: same subject or monozygotic twins; Red: parent/offspring; Blue: full sibling; Green: second degree relative; Brown: third degree relative. Each contour line shows the area that is predicted to include 95% of the sample pairs for each type of relationship.

Fig 5 shows the distribution of HGMR and AGMR values of all pairs of subjects in dbGaP Fingerprinting Collection reported by submitters as closely related. The contour lines show the positions with the same predicted probability densities, and the areas within each contour line are expected to contain 95% of the subject pairs with each relationship type, excluding MZ twins.

### Comparison of GRAF and KING on Determination of Other Types of Relationships

**Fig 6:**
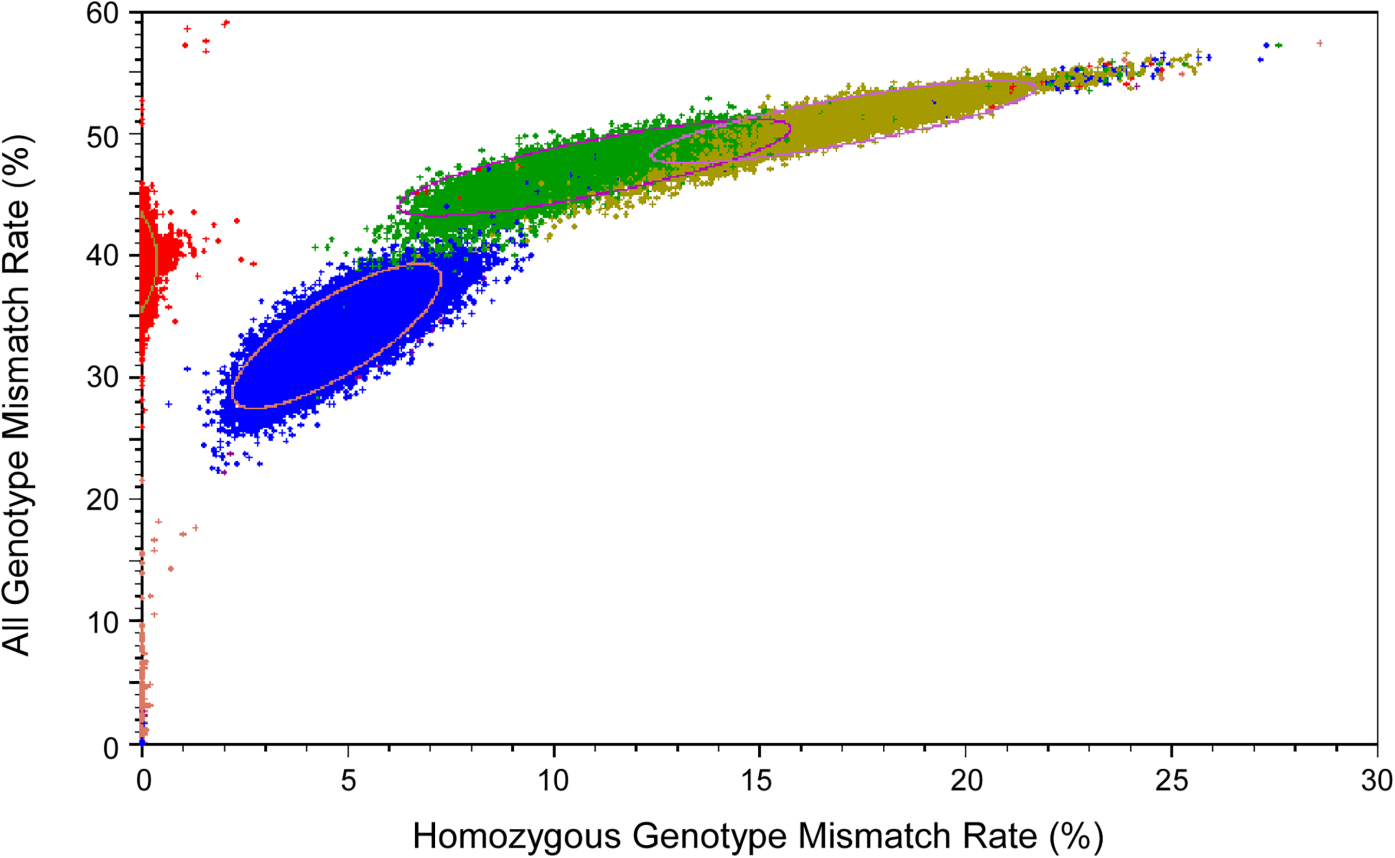
Comparison of GRAF and KING on determining subject relationships for four dbGaP studies. Relationships self-reported in the pedigree files are color coded: red = parent-offspring; blue = full sibling; green = second degree; deep yellow = third degree. Cyan lines show the cutoff values to separate different types of relationships from one another.

We also compared GRAF and KING 2.0 on determining other types of closely related subjects. The same four studies with sufficiently large numbers of different relationships (see Table 6) were used to compare the performances of GRAF and KING 2.0. Fig 6 shows the results of GRAF and KING in scatter plots. For the GRAF results, it plots the AGMR values against HGMR values. For KING results, it plots kinship coefficient estimations against percentages of IBS0 SNPs.

The cyan lines in Fig 6 shows the cutoff lines used by the programs to separate different types of relationships. KING only uses kinship coefficient to separate different relationships, and hence the cutoff lines in the graph are straight lines. The cutoff values shown in Fig 6 are suggested by the authors. However, both PO and FS are first degree relatives, and are expected to have the same kinship coefficients. After first degree relatives are separated from other relationships, KING uses percentage of SNPs with IBS0 to distinguish PO from FS. GRAF uses both HGMR and AGMR to determine the relationships. The cyan cutoff lines in Fig 6 show the show the positions where the probabilities of the two types of relationships, calculated using either Eq 13 or Eq 15, are equal (see Methods).

Fig 6 shows that when distinguishing full siblings from second degree relatives, the HGMR/AGMR combination is better than the combination of IBS0 and kinship coefficient estimation. If only one statistical metric is used to separate full sibling pairs from second degree relatives, HGMR is better than IBS0 and AGMR is better than kinship coefficient estimation.

The above conclusions might not be evident if we only check the plots with visual inspection. We can determine how well the values of two values are separated from each other by using receiver operating character (ROC) curves. The area under a ROC curve (AUC) reflects the discriminatory accuracy of a method. However, ROC curves are useful only when there are some overlaps between the two variables. In the case of these four dbGaP studies, full siblings are very well separated from second degree relatives using any of the four metrics, with no or very little overlaps. It is hard to use ROC curves to evaluate the discriminatory accuracies, since the AUC values will be 1 or very close to 1.

For two independent normal random variables with mean and variances $\mu_{x}$, $\sigma_{x}^{2}$ and $\mu_{y}$, $\sigma_{y}^{2}$, AUC can be calculated using the following equation:

$$AUC=\Phi (\delta ),\delta =\frac{\mu_{x}-\mu_{y}}{\sqrt{\sigma_{x}^{2}-\sigma_{y}^{2}}}$$

where Ф is the standard normal cumulative distribution function[32,33].

The value of *δ* can be estimated using the mean and standard deviations of the HGMR, AGMR, IBS0 and kinship coefficient estimations calculated from the samples being checked. The estimated *δ* values reflect how well two variables are separated. The larger the δ values, the better the two variables are separated. Table 9 shows the *δ* values when different metrics are used to distinguish full siblings from second degree relatives, and second degree relatives from third degree relatives, for the four studies.

**Table 9:** *δ* values when different metrics are used to separate different types of relationships. Kinship = kinship coefficient estimated by KING.

	δ value for full sibling/second degree				δ value for second degree/third degree			
Phs	HGMR	IBS0	AGMR	Kinship	HGMR	IBS0	AGMR	Kinship
phs000763	3.64	3.22	5.62	4.88	2.97	2.97	2.46	2.79
phs000462	3.42	3.08	4.71	4.38	2.64	2.70	2.16	2.45
phs000397	2.85	2.60	4.36	3.83	2.57	2.55	2.25	2.43
phs000168	3.22	2.94	5.07	4.56	2.35	2.47	1.79	2.27

Table 9 shows that when distinguishing full sibling pairs from first degree relatives, HGMR is better than IBS0, and AGMR is better than kinship coefficient estimation. When separating first degree relatives from second degree relatives, HGMR is similar to IBS0, but kinships coefficient estimation is better than AGMR.

### The GRAF software package

We have developed a software package called GRAF (**G**enetic **R**elationship **A**nd **F**ingerprinting) to extract the genotypes of the 10,000 dbGaP fingerprinting SNPs from datasets and to find identical pairs and other closely related subjects. GRAF takes the input for one or more datasets in byte-encoded (a.k.a., binary) PLINK format, along with some auxiliary information that makes it possible to recognize markers by position, dbSNP rs identifier, or dbSNP ss identifier. GRAF also compares the self-reported relationships in the pedigree file with relationships predicted using the genotypes. If multiple datasets are input, then GRAF compares all samples within and across all datasets in a pairwise manner. GRAF implements the quadratic algorithm to find the identical pairs and uses the aforementioned Bayes theorem method to determine subject relationships.

The main program of GRAF is implemented in C++. An auxiliary program for visualizing results is implemented in Perl. Genotypes of every 64 SNPs are encoded and stored in two 64-bit integers, and compared using bitwise operations and multithreading technique to improve the speed (Supplemental Material).

The executable files for GRAF and some documentation can be downloaded from a link at http://www.ncbi.nlm.nih.gov/projects/gap/cgi-bin/Software.cgi. The main program is precompiled to run on 64-bit Linux computers.

### Using GRAF as a QC tool to find errors in data submitted to dbGaP

**Fig 7:**
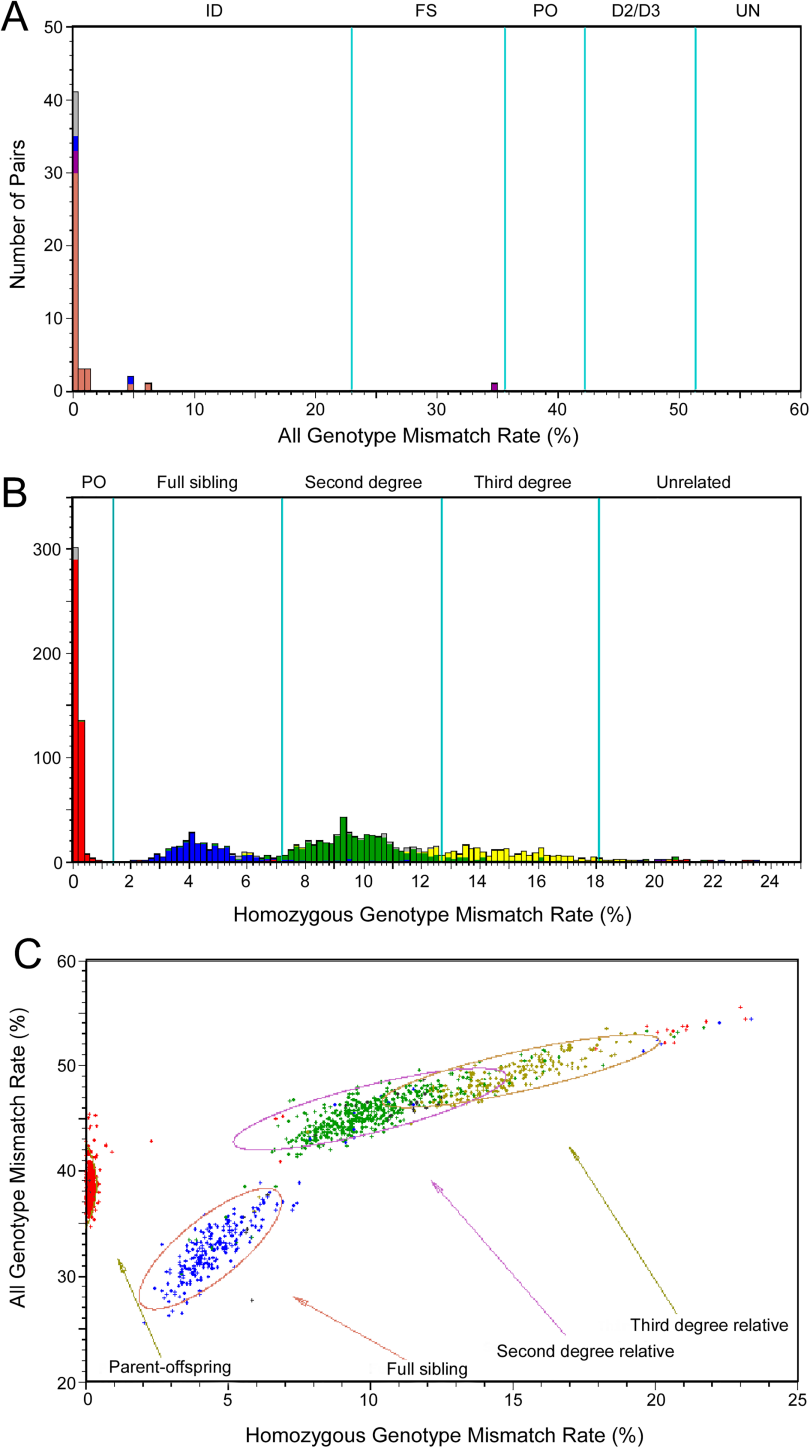
An example of GRAF results displayed on dbGaP website for curators and data submitters to find discrepancies between genotypes and pedigree files submitted to dbGaP. The graphs show GRAF results of one dbGaP study before the errors were corrected by the submitter. Relationships reported by the submitter are color coded. (A) Distribution of AGMR values. Coral red: Duplicate samples; Purple: monozygotic twins; Blue: first, second, or third degree relative; Gray: no relationship reported by submitter. (B) Distribution of HGMR values. Red: parent/offspring; Blue: full sibling; Green: second degree relative; Yellow: third degree relative; Grey: no relationship reported by submitter. (C) Distribution of both HGMR and AGMR values of pairs of related samples, excluding those from same subjects or monozygotic twins. Red: parent/offspring; Blue: full sibling; Green: second degree relative; Yellow: third degree relative; Gray: no relationship reported by submitter.

It is common that the data submitted to dbGaP contain errors, such as misreported subject relationships. For each study submitted to dbGaP, we use GRAF to validate the pedigree file and subject-sample mapping file against the submitted genotype data, and find closely related pairs of subjects that are not reported. The results are made available to the submitters with histograms and scatter plots as well as text tables so that dbGaP curators and data submitters can easily find the potential errors in the submitted datasets. Fig 7 shows the genotype QC results of one dbGaP study with different types of errors. Fig 7A shows that three pairs of subjects that are reported in the pedigree as related but not MZ twins are actually genetically identical, one pair of MZ twins in the pedigree are probably DZ twins, and six pairs of subjects with identical genotypes are not reported in the pedigree and subject-sample mapping file. Fig 7B and 7C show that some subject pairs reported as related in the pedigree file are either related with different types of relationships or unrelated, and some closely related subject pairs are not reported in the pedigree file.

GRAF is used by dbGaP curators and submitters as a quality control tool to check the genotype and pedigree files for all studies. The graphs generated by the GRAF auxiliary program enable users to quickly observe the overall relatedness among subjects, as well as the genotyping error rates. GRAF is also used to find subject overlaps across dbGaP studies and the results are reported to the submitters when appropriate. After subject overlaps are confirmed by the submitters, we assign a single dbGaP ID for multiple samples from the same individual. Users who download data from multiple dbGaP studies can find subject overlaps across dbGaP studies by checking dbGaP subject IDs. To find related, but not identical subjects, users need to rerun GRAF on the datasets downloaded.

For early studies that were released before GRAF was developed, the retrospective checking was limited to identifying duplicate samples and assigning them a shared identifier. For newer studies deposited in dbGaP, we have been requiring that all of the identical pairs should be correctly reported in the subject-sample mapping file and/or pedigree file before the study is released. In practice, all of the discrepancies between the first degree relatives detected by GRAF and those reported in the submitted files must be checked by the submitters; errors in the submitted files should be fixed. If the submitters are not able to fix the errors, we include the GRAF results in README files available to authorized users. If a study has already been released, we ask the submitter to fix the errors in new versions.

## Discussion

We have developed and tested algorithms to quickly determine the subject relationships using SNP genotype data. To date, millions of individuals have been genotyped or sequenced on genome scale using different methods. Before GRAF was developed, there was no computer software available to find duplicate samples in large datasets with samples size as many as a million or more. As of today, besides GRAF, KING 2.0 is the only existing software that can process a dataset with a million samples in a few hours, not days or months. However, KING cannot be used to find duplicate samples obtained using different methods, especially when DNA strand orientations of the same SNPs are different. In addition, KING has not be been tested with large and real genotype datasets when many genotypes are missing or incorrect. We have developed a robust tool to extract genotypes of common SNPs from datasets obtained using different methods and quickly and robustly find all duplicate samples from very large datasets.

For finding duplicate samples and monozygotic twins, we check the genotype discordance rate over all the SNPs (AGMR). When the genotype missing rate is low, one can use the sub-quadratic algorithm to find all the duplicate samples. Depending on the genotype missing and error rate, and the desired prediction precisions and computation times, one can set the parameters accordingly, using equations provided in Methods. GRAF’s quadratic algorithm was able to find about 99.9% of the identical pairs in a combined dataset with 884,794 samples and genotype missing rate up to 90% per sample, when all of the identical pairs missed by the program were those with high genotype discordance rates, and the sensitivities were close to those predicted using the equations in Methods. Many more identical pairs in the same datasets were missed by KING 2.0. KING 2.0 finds identical pairs in several stages. In the first stage it checks genotypes of few SNPs. Then, at the subsequent stages it checks more and more SNPs, until at the last stage, all of the SNPs are checked. This algorithm runs in quadratic time, since usually most of time will be spent on the first stage when the number of SNPs checked is independent of the total number of SNPs genotyped. However, when genotype missing rate is high, since not enough SNPs are checked, one would predict that some of the identical pairs will be missed at the first stage, and will not be found in the subsequent sages. The test results with the real, combined genotype dataset from different dbGaP studies (Table 5) confirmed the above hypothesis. When the genotype missing rate is 30% or more, about 60% of the identical pairs were missed by KING 2.0. So KING 2.0 can only be used to find identical pairs for datasets with low genotyping missing rates, but GRAF quadratic algorithm can robustly tolerate very high genotype missing rate, as long as the total numbers of correctly genotyped SNPs are enough to determine identical pairs.

The quadratic algorithm can find all identical pairs in a dataset with a million samples in a few hours, using current multi-CPU machines. However, when the sample size further increases, e.g., to hundreds of millions samples, the computation time will be days or months. The sub-quadratic algorithm finds identical pairs much faster. However, it does not work for datasets with high genotype missing rates. The sub-quadratic algorithm will be useful for finding identical pairs in datasets with very large sample sizes, or when computer hardware is limited or short running time is desired.

For determining subject relationships, we use the homozygous genotype mismatch rate (HGMR). Since the HGMR is similar to the AGMR, we can use algorithms similar to that for identical pairs to find non-identical but closely related subjects, by adjusting some parameters and the output specifications. However, since many SNPs are ignored when calculating HGMR, which is equivalent to high genotype missing rate for the algorithm, the sub-quadratic algorithm can only be used to find duplicate samples and identical twins.

The ratio of SNPs with IBS state 0 (IBS0) has been used as the basis of published methods to determine subject relationships [11,13,34,35]. Similar to IBS0, HGMR is also calculated with the number of SNPs with IBS state 0. However, IBS0 is the ratio to all the genotyped SNPs, while HGMR is the ratio to the genotyped SNPs with two homozygous genotypes. HGMR is simple enough so that a quadratic algorithm can be implemented to find the related subjects, while it is more sensitive in distinguishing different types of relationships than methods based on IBS0, especially in separating second degree relatives from full siblings (Figs 4 and 5).

Since different types of related subjects are expected to have different probabilities of IBD states (Table 2), most of the existing methods determine the subject relationships by calculating the probabilities of different IBD states[13], or kinship coefficients[11]. The probabilities of different IBD states can be estimated based on the observed IBS states. There are two types of methods to estimate the probabilities of different IBD states using IBS states[23], maximum-likelihood estimation[20] and method-of-moments estimation[22,36]. The practical problems with these methods were summarized in the Introduction.

To determine close genetic relationships between subjects in an outbred population, it is not absolutely necessary to calculate the probabilities of different IBD states, or kinship coefficients. Since for any two subjects, the observed IBS states are dependent on the probabilities of different IBD states, and the latter are determined by the genetic relationship, the prior probability distribution of IBS states can be calculated given a relationship type. The posterior probabilities of subject relationships given certain IBS states can be calculated using the Bayes theorem. However, when inbreeding exists, some parents are themselves closely related and the number of relationship types that need to be distinguished grows. In populations with substantial inbreeding, the relationship types can be much more complex and estimation of kinship coefficient matrix is central to effective genetic analysis[37,38].

The prior probability distributions predicted using homogeneous, random mating population models are considerably different from the real distributions (Fig 4). With large databases, such as dbGaP, one can empirically predict the prior probability distributions, and use these prior distributions to determine the relationships of any pair of subjects. Since no probabilities of IBD states need to be calculated in this process, no values are truncated, and results are not biased.

We use test statistics AGMR and HGMR, derived from IBS states to directly determine subject relationships. For determination of cryptic relationships and identification of pedigree errors, it is very important to be able to distinguish full siblings from second degree relatives since usually it is very easy to separate full siblings from parent-offspring pairs. Fig 6 shows that in distinguishing full siblings from second degree relatives, HGMR is better than IBS0, and AGMR is better than the estimation of kinship coefficient. When GRAF combines HGMR and AGMR together when determining subject relationships, and hence can obtain even higher discrimination power.

AGMR and HGMR are useful in checking pedigree relationships and genotype datasets for quality control. At dbGaP, we calculate the AGMR and HGMR values for all the duplicate samples and closely related subjects, including all the related subjects as reported in the subject-sample mapping file and pedigree file, and plot the distributions of the AGMR and HGMR values. For single datasets, users of GRAF can quickly get the following useful information by examining the graphs visually.

1. The potential errors in the subject-sample mapping file and pedigree file, or sample swapping in the genotype datasets, as indicated by the outliers at each relationship type.
2. The extent of relatedness among subjects as reflected by the numbers of pairs at each relationship type.
3. The genotyping error rate as indicated by the tails of the duplicate sample peak at the AGMR distribution curve and the tails of the parent-offspring curve.

Our analyses and test results above showed that the AGMR and HGMR values for a pair of samples can only be either 0 or much greater than 0, if there are no genotyping errors. For any pairs of samples with AGMR or HGMR slightly greater than 0, all of the genotype mismatches are most likely the result of genotyping errors. If two samples are not duplicates, then a low HGMR value (say less than 2%) indicates that the two subjects are parent-offspring. Empirically, a long tail of the peak near 0 in the AGMR or HGMR distribution curves indicates low genotyping quality.

GRAF can be used to find duplicate samples and identical twins across datasets, even if the genotypes are obtained using different methods. When sufficiently many fingerprint SNPs are covered by the genotyping methods, GRAF can also be used to find the related subjects across different datasets, and verify the pedigree files against the genotypes.

We have applied our algorithms and methods to the quality control checking of datasets submitted to dbGaP and helped the submitters to find and correct inconsistencies in the files. These algorithms and methods can be applied to other datasets. They might also be useful in forensic science for finding subject relationships.

One limitation of GRAF is that it does not take population stratification into consideration. It works very well in determining subject relationships and finding errors in pedigree files with dbGaP studies, where population stratification is not a serious problem. Many computational methods have been developed to find related subjects in admixed populations, e.g., KING-robust[11], REAP[35], PC-relate[34]. We have tried to use the genotypes of the fingerprinting SNPs to predict subject populations. Our preliminary results show that genotypes of the 10,000 fingerprinting SNPs are enough to determine subject populations in sub-continental levels (data not shown). When the method to determine subject populations using fingerprinting SNPs are ready to be used within GRAF, then GRAF can be improved to deal with datasets of admixed populations.

Another limitation is that the implementation of our methods in GRAF requires that at least some of the 10,000 fingerprinting SNPs be genotyped. The more fingerprinting SNPs that are genotyped, the more accurate the algorithm will be. When no or few fingerprinting SNPs are genotyped, no related subjects will be correctly identified. To solve this problem, we could compute the missing genotypes using imputation. In the longer-term, we believe that the genomic community would be better served if genotyping arrays and assays can be standardized, so that they included a common set of SNPs, for example, some or all of the 10,000 fingerprinting collection used by dbGaP. In addition, we will adjust the fingerprint SNP set in future, e.g., adding more SNPs covered by new technologies such as whole exome sequencing, removing some SNPs that were initially determined as bi-allelic but later proved to be multi-allelic.

Scientists who collect or analyze genotype data for GWAS may find GRAF useful in at least three scenarios. First, prospective submitters to dbGaP can run GRAF themselves and streamline the data submission process. If GRAF reports some predicted relationship that the submitter knows to be false, the submitter will be able to alert dbGaP curators of this discrepancy. Second, scientists who wish to do combined analysis of datasets at the dbGaP and EGA repositories may use GRAF to remove duplicate and closely related samples. Third, scientists who are collecting new samples to study some trait can access previous studies for that trait and use GRAF to evaluate whether prospective new study participants participated in another study. The second and third usage scenarios suggest an important area for future development of the GRAF software. It would be helpful to add to GRAF the capability to do only incremental comparisons. As datasets grow, only pairs of individuals in which at least one individual is new, since the last GRAF test, would be compared.

## Methods

### The naïve, quadratic and sub-quadratic algorithms to find identical pairs

Among the relationships in Table 2, it is easiest to distinguish identical pairs from other relationships. Identical pairs are important because they reveal hidden sample duplicates. Since identical pairs have IBD states *Z = 2* at all sites, the observed IBS states will also be *I = 2* at all sites (Table 1) provided there are no genotyping errors, no mutations and no somatic mosaicism. We can use the percentage of SNPs with IBS state *I < 2*, to determine if two samples are genetically identical. Assume a pair of subjects with *m* independent SNPs having the same IBD state either 0, 1 or 2. If we randomly select a SNP, then the conditional probabilities that the two subjects have genotype mismatch (*i*.*e*., *I < 2*) can be calculated using the following equations:

P(I<2|Z=2)=0 (1)

$$P\left(I<2|Z=1\right)=1-\frac{1}{m}\sum_{i=1}^{m}\left(p_{i}^{3}+q_{i}^{3}+p_{i}^{2}q_{i}+p_{i}q_{i}^{2}\right)$$ (2)

$$P\left(I<2|Z=0\right)=1-\frac{1}{m}\sum_{i=1}^{m}\left(p_{i}^{4}+q_{i}^{4}+4p_{i}^{2}q_{i}^{2}\right)$$ (3)

So, of the expected AGMR value for each type of relationship in Table 2 is:

$$P\left(I<2\right)=\sum_{k=0}^{2}P\left(Z=k\right)P\left(I<2|Z=k\right)$$ (4)

If the frequencies of each allele are uniformly distributed in some interval, and if *m* is sufficiently large, then the expected genotype mismatch rates can be calculated using the above equations. The rightmost column of Table 2 shows the predicted mismatch rates. In practice, the 10,000 SNPs were selected with known allele frequencies and those frequencies are not uniform, but we tested and show in the examples below that the approximation works adequately for our data.

To determine whether two samples are genetically identical, we compare the genotypes of these two samples over all the SNPs and calculate AGMR. The complexity of this naïve algorithm is *O(n*^*2*^*S)*, where *n* is number of samples and *S* is the number of SNPs.

Absent genotyping errors, we could conclude that two samples are non-identical if any genotype mismatch is found. Unfortunately, genotyping errors are common with current technologies and mutations do occur rarely. To overcome genotyping errors, we can check more SNPs until a certain number of genotype mismatches are encountered. Suppose that the genotype mismatch rate resulting from genotyping error is *ε*, and we randomly select *m* SNPs with genotypes and set a threshold *k*, such that two samples are deemed non-identical when there are *k* or more genotype mismatches. For that randomized method, the pair-wise identity false negative rate is expected to be:

$$FN=P_{ID:mismatches\geq k}=1-\sum_{i=0}^{k-1}\frac{m!}{\left(m-i\right)!i!}\varepsilon^{i}{\left(1-\varepsilon \right)}^{m-i}$$ (5)

where *P*_*ID*:*ismatches≥k*_ is the probability that there are *k* or more genotype mismatches for an identical pair of samples. In practice, the genotype mismatch rate varies from marker to marker. If the two samples are from two unrelated subjects, then the probability that there is a genotype mismatch at any single SNP is given in Eq 3.

Denote the probability of genotype mismatch for an unrelated pair as *η*, i.e., *η = P(I<2|Z = 0)*, and assume all the non-identical pairs are from unrelated subjects, the false positive rate can be calculated using the following equation:

$$FP=P_{UN:mismatches<k}\approx \sum_{i=0}^{k-1}\frac{m!}{\left(m-i\right)!i!}\eta^{i}{\left(1-\eta \right)}^{m-i}$$ (6)

In practice, the sample pairs are not all independent, since each sample is compared against all other samples. However, for sufficiently large sample size, the above estimates of false negative and false positive rates should be accurate.

Eqs 5 and 6 show that we can implement an algorithm to check only a subset of the SNPs to find all the identical pairs (ID in Table 2). Since the number of SNPs that need to be checked (*m*) is not dependent on the total number of genotyped samples (*n*), the complexity of this algorithm is *O(n*^*2*^*)*. This algorithm is referred to as the “quadratic algorithm”.

The identity false positive rate can be minimized by checking the genotypes of all the *S* SNPs for those pairs having fewer than *k* mismatches out of *m* SNPs. If the false positive rate calculated using Eq 6 is low and there are relatively few identical pairs, then checking genotypes of more SNPs as mentioned above will not dramatically increase the running time.

Since the genotypes of every 64 SNPs are stored in two 64-bit integers, in the GRAF software, the quadratic algorithm is implemented by setting *m* = 64 and *k* = 8, i.e., the program stops checking genotypes if 8 or more genotype mismatches are found after 64 SNPs with non-null genotypes are compared. If the genotype mismatch rate *ε* resulting from genotyping error is known, we can predict the sensitivity of the quadratic algorithm using Eq 5. Since the program keeps on checking genotypes until enough number of non-null genotypes are compared, the quadratic algorithm works even if the genotype missing rate is high.

If the genotype missing and error rates are both low, we can use a faster algorithm to find all the identical pairs. When all SNPs are correctly genotyped for all the samples, we can randomly select an ordered list of SNPs and save the genotypes of each sample in a string or integer. Then, we sort all samples by their genotypes and walk through the sorted list to find the identical pairs, which will appear next to each other on the list. The complexity of this sub-quadratic algorithm will be *O(n log n)*, which is the complexity of the sorting step.

However, if some genotypes are missing or incorrect, some of the identical pairs will not be consecutive on the list, and hence will be missed by this algorithm. For each identical pair, if we randomly select sets of *m* SNPs, the numbers of SNPs with missing or mismatched genotypes should roughly follow a Poisson distribution. Let *δ* be the rate of SNPs with missing or mismatched genotypes, the probability that none of the *m* SNPs have missing or mismatched SNPs is equivalent to the sensitivity of this method, which can be calculated as:

$$Sensitivity=P_{all\_matches}=e^{-m\delta }$$ (7)

To increase the sensitivity of the sub-quadratic algorithm, we can randomly select another set of SNPs with replacement and repeat the sorting and comparison of adjacent items. Assuming the missing or incorrect genotypes are randomly distributed among different samples and SNPs, if we repeat the process for *r* rounds, then the false negative rate of this method will be:

$$FN={\left(1-e^{-m\delta }\right)}^{r}$$ (8)

A more structured description of the sub-quadratic algorithm is as follows:

Sort SNPs by genotype missing rate, from low to high

Putative ID pair set = {}

Real ID pair set = {}

Loop until *done*

Randomly select *m* SNPs from the top of list

Code the genotype of each sample with an integer

Sort the samples by these integers

Check the sorted list to find new putative ID pairs

Add new putative ID pairs to the putative ID pair set

For each pair among the new putative ID pairs

Check all the 10,000 SNPs to determine if they are ID

Add new real ID pairs to the real ID pair set

*done* if no new real ID pair found in *c* consecutive rounds

The number of SNPs to check each time (*m*), and number of consecutive rounds without new ID pairs (*c*) should be selected based on the requirements of sensitivity and running time, as well as the quality of the genotype datasets. Eq 7 shows that the sensitivity of each round decreases when *m* increases. However, the false positive rate will also increase when *m* increases, which means more time will be spent on checking more SNPs to exclude these extra false negatives. Similarly, increasing the *c* number will decrease the false negative rate, according to Eq 8, at the cost of longer running time.

### Using HGMR to determine other common relationships as non-identical related pairs

It is harder to distinguish non-identical relationships from each other than to separate identical pairs. Table 2 shows that different types of relationships have different *P(Z)* and *P(I)* values. Most of the existing methods determine the relationships by estimating the *P(Z)* values using the observed IBS states. For example, Purcell et al[13] use the observed number of SNPs with IBD state 0, *N(I = 0)*, to estimate *P(Z = 0)* value. For a pair of subjects, if we check *S* SNPs, then the probability to have IBD state *I* = 0 for any SNP can be calculated using the following equation:

$$P\left(Z=0\right)=\frac{P\left(I=0\right)}{P(I=0|Z=0)}$$ (9)

By implementing the method-of-moments approach, some researchers use the *observed* number of SNPs with *I = 0* in replacement of the *expected* number, and estimate the probability as:

$$P(Z\;=\;0)\approx \;\frac{N(I\;=\;0)/S}{P(I\;=\;0|Z\;=\;0)}$$

However, since the *N(I = 0)/S* values, i.e., observed rates of *I = 0* SNPs, can be greater than the predicted probabilities, the calculated values can be larger than 1.

If we only need to determine closely related subject pairs, e.g., up to third degree relatives, we do not have to estimate the *P(Z)* values. Table 2 and Eq 9 show that different non-identical relationships have different expected rates of *I = 0* SNPs. Therefore, we can use the rate of *I = 0* SNPs, without truncating the values, to distinguish these relationships.

A different test statistic, which we call the *homozygous genotype mismatch rate* (HGMR), can also be used to separate closely related non-identical pairs, with discrimination power higher than the rate of *I = 0* SNPs, especially in separating full sibling pairs from second degree relatives. For each pair of samples, we define HGMR as the genotype mismatch rate when only homozygotes are considered. Let A and B be the two alleles of any one SNP, we count only the SNPs with the following genotypes: either same homozygotes (SO, equivalent to genotypes AA|AA and BB|BB) or different homozygotes (DO, equivalent to genotypes AA|BB and BB|AA), and calculate HGMR using the following equation:

$$HGMR=\frac{N_{DO}}{N_{DO}+N_{SO}}=\frac{N_{AA|BB}+N_{BB|AA}}{N_{AA|BB}+N_{BB|AA}+N_{AA|AA}+N_{BB|BB}}$$ (10)

The different homozygotes (DO) state implies the IBS state *I = 0*, i.e., *N*_*DO*_ = *N(I = 0)*, but the same homozygotes (SO) state does not imply the IBS state *I = 2*.

Let *p*_*i*_ and *q*_*i*_ be the frequencies of the two alleles for one SNP. The probabilities to have different homozygotes and same homozygotes can be computed using the following equations:

$$P_{i}(DO)=P(Z=0)P_{i}(DO|Z=0)$$

$$P_{i}(SO)=\sum_{k=0}^{2}P(Z=k)P_{i}(SO|Z=k)$$

where the conditional probabilities can be derived from Fig 1:

$$P_{i}(DO|Z=0)=2p_{i}^{2}q_{i}^{2}$$

$$P_{i}(SO|Z=0)=p_{i}^{4}+q_{i}^{4}$$

$$P_{i}(SO|Z=1)=p_{i}^{3}+q_{i}^{3}$$

$$P_{i}(SO|Z=2)=p_{i}^{2}+q_{i}^{2}$$

If we check *S* SNPs for subject pairs, *N*_*DO*_ and *N*_*SO*_ values will each roughly follow a binomial distribution, and the expected HGMR value will be:

$$E(HGMR)\approx \frac{\sum_{i=1}^{S}P_{i}(DO)}{\sum_{i=1}^{S}\left[P_{i}(DO)+P_{i}(SO)\right]}$$ (11)

The expected value of the quotient of two random variables is not the same as the quotient of the two expected values. However, in this case, all the values are positive and the coefficient of the variables in the denominator is small, so the expected quotient is close to the quotient of the two expected values. In addition, since usually *N*_*SO*_ is much greater than *N*_*DO*_, the standard deviation of HGMR is mostly dependent on the deviation of the numerator *N*_*DO*_, and hence, can be estimated using the following equation:

$$SD(HGMR)\approx \frac{\sqrt{P_{DO}(1-P_{DO})S}}{\sum_{i=1}^{S}\left[P_{i}(DO)+P_{i}(SO)\right]}$$ (12)

where *P*_*DO*_ is the probability that on a random SNP location the two subjects have different homozygous genotypes:

$$P_{DO}=\sum_{i=1}^{S}P_{i}(DO)/S$$

When allele frequencies are available, we can predict the mean HGMR values and standard deviations for any type of relationships using the above equations. Table 3 shows the predicted HGMR values and standard deviations for different types of relationships, assuming allele frequencies are uniformly distributed over a range of 0.1 to 0.9, and sufficiently large amount of independent SNPs are compared.

### Using both HGMR and AGMR to determine subject relationships

As described above, for pairs of samples, the numbers of SNPs with matched and mismatched genotypes are expected to follow binomial distributions. Therefore, when the number of SNPs compared is sufficiently large, the HGMR and AGMR values of each relationship type, except for parent-offspring and identical pairs, should approximately follow normal distributions, and the combination of both variables should follow a bivariate normal distribution. In general, if *x* and *y* are two variables following normal distributions

$$x\;∼\;N(\mu_{x},\;\sigma_{x}^{2}),$$

$$y\;∼\;N(\mu_{y},\;\sigma_{y}^{2}),$$

then we have the following probability density function for both *x* and *y*:

$$P\left(x,y\right)=\frac{1}{2\pi \sigma_{x}\sigma_{y}\sqrt{1-\rho^{2}}}\exp\left(\frac{-1}{2\left(1-\rho^{2}\right)}\left[\frac{{\left(x-\mu_{x}\right)}^{2}}{\sigma_{x}^{2}}-\frac{2\rho \left(x-\mu_{x}\right)\left(y-\mu_{y}\right)}{\sigma_{x}\sigma_{y}}+\frac{{\left(y-\mu_{y}\right)}^{2}}{\sigma_{y}^{2}}\right]\right)$$ (13)

where *ρ* is the correlation coefficient between *x* and *y*:

$$\rho \;=\;\frac{covariance(x,y)}{\sigma_{x}\sigma_{y}}$$

If we set *x* = HGMR and *y* = AGMR and estimate the values of *μ*_*x*_, *σ*_*x*_, *μ*_*y*_, *σ*_*y*_, and *ρ* of the whole population of each relationship type using the genotype data of a set of subjects sampled from the population, we can use the above equations to determine the relationship between any pair of subjects in the population.

Eq 13 can be used to calculate the probability to get a specific pair of HGMR and AGMR when a subject pair is known to have relationship full sibling (FS), second degree relative (D2), third degree relative (D3), or unrelated (UN). However, since the HGMR values of parent-offspring (PO) pairs are not normally distributed, the above equation is not applicable to PO pairs.

For PO pairs, if there are no genotyping errors, HGMR values are expected to be 0. The homozygous genotype mismatches of PO pairs are all caused by genotyping errors or the rare mutational event. If genotyping errors were random over all the SNPs, then HGMR values would follow a Poisson distribution. Unfortunately, the actual genotyping errors are not random and the HGMR values of PO pairs do not follow a Poisson distribution. For subjects in dbGaP, the number of PO pairs roughly decreases exponentially when HGMR increases. We use the following exponential distribution function to estimate the distribution of HGMR values of PO pairs:

$$P_{PO}\left(x\right)=ke^{-kx}$$ (14)

where *x* ≥ 0.

We have

$$\int_{0}^{\infty }ke^{-kx}\;dx\;=\;1.$$

The value of *k* is empirically set based on the actual HGMR values of all the pairs reported as PO in the files submitted to dbGaP. Since usually, it is easy to distinguish PO pairs from other relationships, *k* can be selected in a wide range without affecting the accuracy of genetic relationship prediction.

Let us also assume that for PO pairs HGMR and AGMR are independent, i.e., with correlation coefficient 0. Since AGMR values of PO pairs follow a normal distribution, we can use the following probability density function to estimate the distribution of both HGMR and AGMR values of PO pairs:

$$P_{PO}\left(x,y\right)=\frac{k}{\sigma_{y}\sqrt{2\pi }}e^{-kx-\frac{{\left(y-\mu_{y}\right)}^{2}}{2\sigma_{y}^{2}}}$$ (15)

The above simple model can only roughly estimate the probability distribution of HGMR and AGMR values of PO pairs. Fortunately, since PO pairs can be well separated from other relationship types using HGMR and AGMR (see analyses above), this simple model can still be used to distinguish PO pairs from other relationships.

When AGMR and HGMR probability density functions are available for all relationships, we can use them to determine the relationship of any pair of subjects. Given a set of subjects with genotypes, sampled from the same homogeneous population, we first calculate the HGMR and AGMR values for every pair of samples. Second, we use a cutoff AGMR value to exclude identical pairs, and a cutoff HGMR value to exclude all the remotely related and unrelated pairs. Third, suppose all the subject pairs remaining after the second stage have relationships in {PO, FS, D2, D3}, denoted by *T*. For each pair of subjects, if the HGMR and AGMR values are *x* and *y*, respectively, the probability that this pair has relationship *R* ∈ *T* can be calculated using the following equation:

$$P(R|xy)=\frac{P(R)P(xy|R)}{\sum_{t\in T}P(t)P(xy|t)}$$

If we also assume that the *P(t)* values are the same for all *t* ∈ *T*, then the above equation becomes:

$$P(R|xy)=\frac{P(xy|R)}{\sum_{t\in T}P(xy|t)}$$ (16)

The mean HGMR and AGMR values *μ*_*x*_ and *μ*_*y*_, and the standard deviations *σ*_*x*_, *σ*_*y*_ and correlation coefficient *ρ* are required when calculating the *P(R|xy)* values. Within each dbGaP study, if there are sufficiently many subjects genotyped, we can calculate the allele frequencies for all of the 10,000 fingerprinting SNPs, and then use Eqs 4 and 11 to compute *μ*_*x*_ and *μ*_*y*_ values. In practice, we calculate the standard deviations and correlation coefficients of HGMR and AGMR for all subject pairs in dbGaP with known relationships for each relationship *R* (Table 7) and use these values to estimate *σ*_*x*_, *σ*_*y*_ and *ρ* values empirically. When *μ*_*x*_, *σ*_*x*_, *μ*_*y*_, *σ*_*y*_, and *ρ* values are available for each study, we check every pair of subjects and calculate the *P(R|xy)* values for all relationships *R ∈* {PO, FS, D2, D3}, and determine the relationship as the type that has the maximum *P(R|xy)* value.

### Algorithms to find non-identical but closely related subjects

Using HGMR values, the subject relationships can be determined with algorithms very similar to the algorithm described above for finding identical pairs. For determining identical pairs, the mismatch rates of all genotypes are calculated. When checking for non-identical relationships, we ignore the heterozygous SNPs and calculate the genotype mismatch rates. The quadratic algorithm can be modified to find non-identical related pairs as explained above and in the Supplementary Material. Actually, when SNPs with heterozygous genotypes are skipped for each pair of subjects, finding PO pairs is the same as finding identical pairs, since a PO pair is expected to have no mismatched genotypes, like an identical pair. The only difference is that fewer SNPs are used when finding PO pairs. If we use *m* to represent number of SNPs with valid genotypes, (i.e., those with two homozygous genotypes for one pair of subjects), then the false positive rate can be calculated using Eq 5. Since many SNPs are not valid for calculating HGMR, more SNPs need to be checked each time, and the cutoff value *k* also needs to be adjusted based on the estimation of number of valid SNPs.

## Supporting information

S1 Table Sample sizes and genotype missing rates of studies in the dbGaP fingerprinting collection. (XLSX)

S1 File Bitwise operations to calculate HGMR and AGMR values. (DOCX)
