# Supplementary material for: Quickly identifying identical and closely related subjects in large databases using genotype data
Source: PLoS One. 2017 Jun 13;12(6):e0179106. doi: 10.1371/journal.pone.0179106 (PMC5469481; doi:10.1371/journal.pone.0179106)
Supplement: S1 File — (DOCX) [file pone.0179106.s002.docx]

**Bitwise operations to calculate HGMR and AGMR values**

For each pair of subjects, one can compare genotypes of a certain number of SNPs, ignoring any SNP on which at least one of the genotypes is missing. Denote:

*N_D_*: number of SNPs where the two genotypes are known and different

*N_S_*: number of SNPs where the two genotypes are known and the same

*N_DO_*: number of SNPs where the two genotypes are different and both genotypes are homozygous

*N_DX_*: number of SNPs where the two genotypes are different and at least one genotype is heterozygous

*N_SO_*: number of SNPs where the two genotypes are same and both genotypes are homozygous

*N_SX_*: number of SNPs where the two genotypes are same and heterozygous

The AGMR and HGMR values can calculated using the following equations:

$AGMR= \frac{N_{D}}{{N_{D}+N}_{S}}= \frac{N_{DO}+N_{DX}}{N_{DO}+ N_{DX}+ N_{SO}+ N_{SX}}$

$HGMR= \frac{N_{DO}}{N_{DO}+ N_{SO}}$

Therefore, AGMR and HGMR values can be calculated after the four counts *N_DO_*, *N_SO_*, *N_DX_*, *N_SX_* are available. To obtain these counts, we first code genotypes of every 64 SNPs with two long integers. For example, if the genotypes of one subject at four markers and beyond are AA,BB,OO,AB,…, where A is the reference allele and B is the alternative allele, and O stands for missing genotype, then we code the genotypes using the following two integers:

0110…

0101…

The genotype of each SNP is coded by the two bits from both integers from the corresponding position in the numbers, where 00, 11, and 0l stand for homozygous references, homozygous alternatives, and heterozygous genotype, respectively, and 10 stands for missing genotype. The first row denotes the first alleles and the second row denotes the second alleles. The heterozygous genotype is always denoted AB in letters and 01 in numbers, which is why we can reserve 10 for the unknown genotype.

Let *i_1_*, *i_2_* be the two bits coding genotypes of subject *i*, and *j_1_*, *j_2_* be the two numbers coding genotypes of subject *j*. First we determine whether two genotypes are both homozygous or not (*O* = both homozygous; *X* = not both homozygous):

*isOi = ~( i_1_ ^ i_2_)*

*isOj = ~( j_1_ ^ j_2_)*

*isXi = ~ i_1_ & i_2_*

*isXj = ~ j_1_ & j_2_*

where *isOi, isOj, isXi, isXj* represent whether subject *i* is homozygous, *j* is homozygous, *i* is heterozygous, *j* is heterozygous, respectively, in terms of the genotype at a certain SNP position. Then we compare the genotypes of the two subjects and determine whether or not they are the same:

*isDo = (i_1_ ^ j_2_) & isOi & isOj*

*isSo = ~(i_1_ ^ j_2_) & isOi & isOj*

*isDx = (isOi & isXj) | (isOj & isXi)*

*isSx = isXi & isXj*

The four counts *N_DO_*, *N_SO_*, *N_DX_*, *N_SX_*, which are needed for calculating AGMR and HGMR values, can be obtained by counting the numbers of bits with *isDo, isSo, isDx, isSx* equal to 1. We use the built-in function *__builtin_popcountl* provided by GCC to count the numbers of bits.

We implemented the above method as well as a method that compares one SNP at a time. The method using bitwise operations was approximately 20 times faster when we used 10,000 SNPs in GRAF.
